# Health information exchange policy and standards for digital health systems in africa: A systematic review

**DOI:** 10.1371/journal.pdig.0000118

**Published:** 2022-10-10

**Authors:** Adane L. Mamuye, Tesfahun M. Yilma, Ahmad Abdulwahab, Sean Broomhead, Phumzule Zondo, Mercy Kyeng, Justin Maeda, Mohammed Abdulaziz, Tadesse Wuhib, Binyam C. Tilahun

**Affiliations:** 1 Department of Computer Science, College of Informatics, University of Gondar, Gondar, Ethiopia; 2 Department of Health Informatics, College of Medicine and Health Sciences, University of Gondar, Gondar, Ethiopia; 3 Health Information Systems Program–SA, Pretoria, South Africa; 4 Africa Centers for Disease Control and Prevention, African Union Commission, Addis Ababa, Ethiopia; 5 Centers for Disease Control and Prevention, National Center for Public Health Informatics, Atlanta, Georgia, United States of America; UCSF: University of California San Francisco, UNITED STATES

## Abstract

Lack of interoperability and integration between heterogeneous health systems is a big challenge to realize the potential benefits of eHealth. To best move from siloed applications to interoperable eHealth solutions, health information exchange (HIE) policy and standards are necessary to be established. However, there is no comprehensive evidence on the current status of HIE policy and standards on the African continent. Therefore, this paper aimed to systematically review the status of HIE policy and standards which are currently in practice in Africa. A systematic search of the literature was conducted from Medical Literature Analysis and Retrieval System Online (MEDLINE), Scopus, Web of Science, and Excerpta Medica Database (EMBASE), and a total of 32 papers (21 strategic documents and 11 peer-reviewed papers) were selected based on predefined criteria for synthesis. Results revealed that African countries have paid attention to the development, improvement, adoption, and implementation of HIE architecture for interoperability and standards. Synthetic and semantic interoperability standards were identified for the implementation of HIE in Africa. Based on this comprehensive review, we recommend that comprehensive interoperable technical standards should be set at each national level and should be guided by appropriate governance and legal frameworks, data ownership and use agreements, and health data privacy and security guidelines. On top of the policy issues, there is a need to identify a set of standards (health system standards, communication, messaging standards, terminology/vocabulary standards, patient profile standards, privacy and security, and risk assessment) and implement them throughout all levels of the health system. On top of this, we recommend that the Africa Union (AU) and regional bodies provide the necessary human resource and high-level technical support to African countries to implement HIE policy and standards. To realize the full potential of eHealth in the continent, it is recommended that African countries need to have a common HIE policy, interoperable technical standards, and health data privacy and security guidelines. Currently, there is an ongoing effort by the Africa Centres for Disease Control and Prevention (Africa CDC) towards promoting HIE on the continent. A task force has been established from Africa CDC, Health Information Service Provider (HISP) partners, and African and global HIE subject matter experts to provide expertise and guidance in the development of AU policy and standards for HIE. Although the work is still ongoing, the African Union shall continue to support the implementation of HIE policy and standards in the continent. The authors of this review are currently working under the umbrella of the African Union to develop the HIE policy and standard to be endorsed by the head of states of the Africa Union. As a follow-up publication to this, the result will be published in mid-2022.

## Author summary

With the increased deployment of digital health technologies in healthcare settings across the globe, Health Information Exchange (HIE) is getting increasing attention. This is because it helps healthcare organizations to interchange data, especially at times of pandemics and improves patient safety, efficiency, cost-effectiveness, and quality of health care delivery. However, there is limited evidence on the level of adoption and deployment of HIE systems in many middle- and low-income countries including Africa. In this review paper, we summarized the available evidence and provide comprehensive evidence on the level of HIE adoption in Africa. We found that many African countries outlined several initiatives to develop and integrate digital health systems in their strategic documents. They have also proposed the development, improvement, adoption, and implementation of HIE architecture, interoperability, and standards for HIE. Based on the summary of this evidence, the team worked with Africa CDC to develop an African Union (AU) HIE Policy and Standards document to guide the development of HIE in Africa. In the future, we aim to publish the AU HIE Policy and Standards recommendation document as a follow-up paper that can be used by policymakers as well as researchers.

## 1. Introduction

Digital health has gained a lot of attention globally, especially in the past five years as an engine for innovation to address sustainable development goals (SDGs) and universal health coverage (UHC) [1]. Substantial investments in digital solutions in recent years are significantly improving health services in Africa. Currently, several applications are implemented in Africa ranging from electronic surveillance and digital health applications to telemedicine applications. The widely-used digital health applications in Africa are District Health Information System 2 (DHIS 2) and Open Medical Record System (OpenMRS). DHIS 2 is an open-source web-based software platform for data collection, management, and analysis, and is implemented in 42 African countries [2]. OpenMRS is implemented in facilities of 19 African countries [3]. Clinical data from OpenMRS is increasingly used to inform public health decisions. The use of Telemedicine has highly improved the quality of patient treatment in rural Africa by ensuring access to specialist knowledge in a time of need [4]. Telemedicine provides the following healthcare services in Africa: neonatal care; maternal and child healthcare; intensive-care services; trauma care; occupational healthcare; mental health services; geriatric medicine; nutritional health; radiological services and e-pharmacy services [5]. Mobile health (mHealth) applications, such as Vula [6], MomConnect [7], WelTel [8] and Omomi [9], are promising applications for telemedicine services. These applications have transformed the practices of public health research, disease surveillance and health care delivery in Africa. The manifestation of the Coronavirus Disease 2019 (COVID-19) pandemic has significantly expanded the need and use of digital health solutions in Africa and beyond. Many countries in Africa, for instance, are using DHIS 2 tracker in response to COVID-19.

Despite the progress made in the use of electronic tools and digital health technologies, most of the available solutions are web and mobile-based health systems utilizing technologies that are local, proprietary, and siloed [10]. As a result, health data remains disintegrated with multiple parallel reporting channels with the inability to efficiently track health attributes across the whole health sector and hence affecting the quality of evidence needed for decision making [11]. Lack of a well-designed policy and standards on health information exchange (HIE) that dictate the interoperability between heterogeneous systems has been recognized as a key obstacle to realizing the potential benefits of eHealth in Africa [12]. According to the World Health Organization (WHO) and International Telecommunication Union (ITU) National eHealth Strategy Toolkit, policy compliance, standards and interoperability were considered an enabling environment for national eHealth implementation [13].

To best move from siloed applications to interoperable national digital health ecosystems, HIE policy and standards are necessary. The policy aspect sets out rules and procedures to govern the implementation of HIE among eHealth systems. The HIE standards aspect defines syntax and structure to operationalize data exchange and have robust and interoperable solutions. Interoperability standards in eHealth are broadly categorized as organizational (process, procedures and alignment), syntactic (structure, content and services), semantic (vocabulary, code sets, terminology), and foundational/technical (transport) interoperability [14]. Together, these standards facilitate a secure, seamless, and timely communication and use of data both within and between countries, organizations, entities and individuals.

Successful implementation of HIE policy and standards for two or more eHealth systems can improve patient safety through the provision of the right information about the right patients in a secure manner. For example, Kaelber and Bates state that 18% of patient safety errors and 70% of adverse drug events could be eliminated if the right information about the right patient is available [15] which can be achieved through sound HIE policy and standards. Another outcome of having HIE policy and standards is assuring patients’ and stakeholders’ expectations about privacy and security concerns [16]. Patients and stakeholders are likely not to worry about the confidentiality and security of their health records during data exchange if there are HIE policies and standards that ensure privacy and security.

In Africa, there are few attempts to implement HIE [12,17]. However, currently, no evidence exists indicating the presence of HIE policy and standards in the African continent. There is an initiative from Africa CDC to develop HIE policy and standards for eHealth systems for the AU Member States. Hence, reviewing available literature and providing recommendations is very essential for the development and successful implementation of HIE policy and standards in Africa. Therefore, this paper aimed at systematically reviewing and exploring the existence of HIE policies and standards in Africa to guide the development and implementation of comprehensive and robust health information systems across the continent.

## 2. Methods

**Fig 1:**
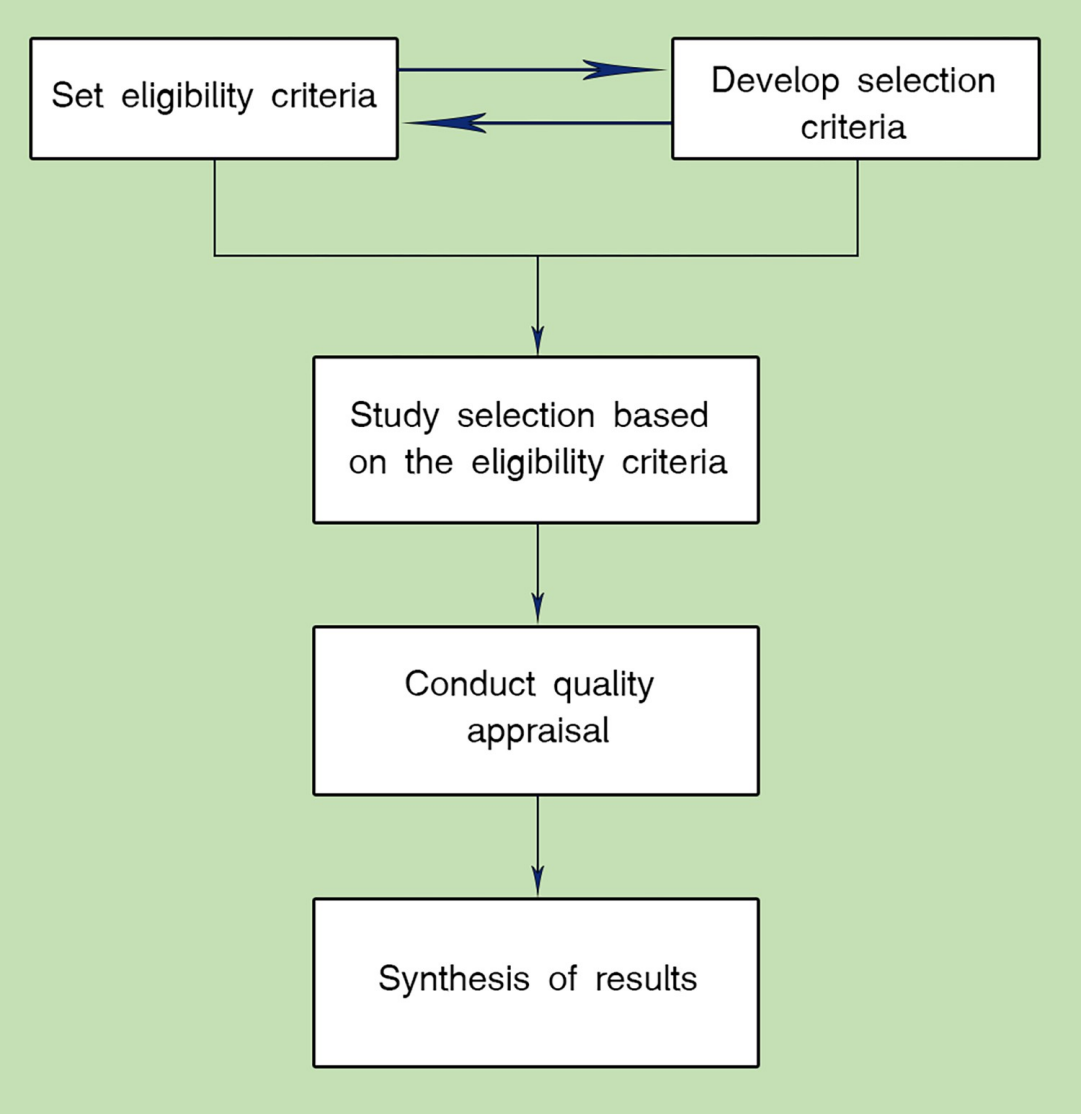
This flowchart shows the methodology followed in the systematic review.

We conducted a systematic literature review as per the block diagram depicted in Fig 1.

*Eligibility Criteria*: This review targeted peer-reviewed publications, governmental reports, and strategy documents related to HIE policy and standards in African countries. We only included publications published in English and between 01 January 2014 and 12 August 2020 in order to capture evidence from the most recent publications.

*Search Strategies*: In order to find relevant publications related to HIE in Africa, major electronic databases of peer-reviewed journal articles, such as MEDLINE, Scopus, Web of Science, and EMBASE were selected. In addition, Google and Google Scholar were used to retrieve governmental documents related to HIE in Africa. Furthermore, peer-reviewed publications were identified from reference lists of relevant reviewed articles that met the inclusion criteria and these articles were retrieved using Google Scholar.

A systematic and comprehensive search was performed by two independent reviewers. The search strategy was developed after identifying keywords and combining them with Boolean operators (“AND” and “OR”). Table 1 shows the main search terms and the alternative keywords. The search terms were combined using Boolean operators to search for potential publications in relation to HIE policy and standards in Africa.

**Table 1:** Search terms of Health information Exchange Policy, Standards, or implementation challenges in Africa.

Main terms	Health Information Exchange	Policy	Standards	Implementation Challenges	Africa
**Alternative words/phrases**	Interoperability	Strategy	architecture	Barriers	Names of all African countries
	Electronic health information exchange	Framework	Model	Problems	
	Electronic healthcare information exchange	Guideline	Protocols	Difficulties	
	Health care information exchange	Recommendation		Bottlenecks	
	Healthcare information exchange	Roadmap			
		Principles			
		Plan			
		Program			
		Action plan			

The final search strategy that was used to search in search engines and database is [(“Health information exchange” OR Interoperability OR “electronic health information exchange” OR “electronic healthcare information exchange” OR “Health care information exchange” OR “Healthcare information exchange”) AND (Policy OR Strategy OR framework OR guideline OR recommendation OR roadmap OR principles OR plan OR program OR “action plan” OR Standards OR architecture OR model OR protocols OR Implementation OR Challenges OR Barriers OR Problems OR Difficulties OR bottlenecks) AND (Africa OR Algeria OR Angola OR Benin OR Botswana OR “Burkina Faso” OR Burundi OR Cameroon OR “Cape Verde” OR “Central African Republic” OR Chad OR Camoros OR Comoros OR “Democratic Republic of the Congo” OR “Democratic Republic of Congo” OR Congo OR “Republic of the Congo” OR “Republic of Congo” OR Djibouti OR Egypt OR “Equatorial Guinea” OR Eritrea OR Ethiopia OR Gabon OR Gambia OR Ghana OR Guinea OR “Guinea Bissau” OR “Guinea-Bissau” OR “Ivory Coast” OR “cote d’ivoire” OR Kenya OR Lesotho OR Liberia OR Libya OR Madagascar OR Malawi OR Mali OR Mauritania OR Mauritius OR Morocco OR Mozambique OR Namibia OR Niger OR Nigeria OR Rwanda OR “Sao Tome and Principe” OR Senegal OR Seychelles OR “Sierra Leone” OR Somalia OR “South Africa” OR “South Sudan” OR Sudan OR Swaziland OR Tanzania OR Togo OR Tunisia OR Uganda OR Zambia OR Zimbabwe)].

*Study selection*: Based on the inclusion and exclusion criteria, all searched records were transferred to Endnote X9, a reference management software, to discard duplicate studies. After duplications were removed, two reviewers independently read the titles and abstracts of the remaining articles to identify both potentially eligible articles and any articles for which a determination could not be made from the title and abstract alone. Then, the selected full text of the remaining articles were examined for eligibility. All disagreements between the reviewers were resolved through consensus.

*Quality appraisal*: A discussion between two independent reviewers was made prior to commencing the quality appraisals by using a random sample of 3 articles. Then, a quality appraisal was performed based on the problem statement, objective, method, citation, result usefulness, and result applicability of the articles, as shown in Appendix A in S1 File. The quality of each article was measured using a 3-point scale (high, moderate and low). High-quality publications are those which have clearly defined objectives, proper citations, adequately described methods and useful results. Moderate quality publications are those whose objective, method and results are inadequately described while low quality is given for publications where any of the quality measures are missed. All disagreements in the quality of the articles between the reviewers were resolved through consensus.

*Synthesis of results*: A data extraction form was developed prior to synthesizing the results. The form included authors’ names, year of publication, objectives, methods, and findings related to HIE policy and standards for peer-reviewed articles while a different format was used for government reports and eHealth strategic documents, see Appendix B and Appendix C in S1 File.

## 3. Results

### 3.1. Study Selection

**Fig 2:**
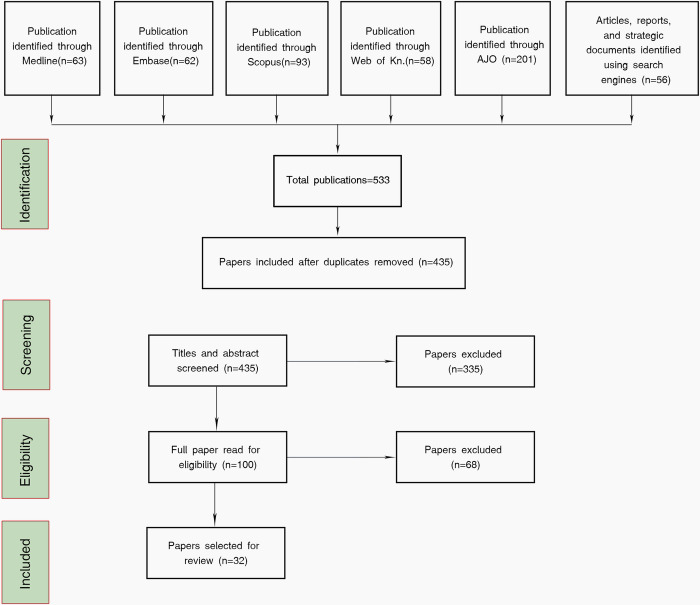
Search results and study selection flowchart.

A total of 533 citations were identified through a comprehensive search from databases and search engines from which 98 of them were duplicates. A total of 403 were excluded based on the eligibility criteria previously outlined and 32 were included in the review (Fig 2).

### 3.3. Characteristics of Studies

Out of the 32 articles reviewed, 11 were journal articles and the remaining 21 were governmental eHealth-related strategy and policy documents. HIE policy-related articles were 23 while HIE standard-related articles were 15 and six articles addressed both HIE policy and standards. Most of the articles (47%) have a moderate quality while only 22% have high quality in terms of their methods, credibility of their citation, and applicability (Appendix A and Appendix C in S1 File). In terms of the study methods, most (55%) of the publications were based upon situational analysis [18–29] using document reviews (e.g., World Health Organization (WHO) eHealth strategy development toolkit), stakeholder interviews (11%), discussion in meetings and workshops (26%), and survey and site visit (8%). Among the journal articles (11), three articles followed interoperable system design and development method [30–32]; another three articles followed framework design [33–35] in which two of them supplemented framework development with a qualitative approach (case study) [34,35]; three articles followed literature review [4,36,37]; and one article followed a consultative workshop for a concept dictionary implementation in Kenya [38] while the remaining one followed a discussion of a specific interoperable platform.

### 3.4. HIE Policies

A total of 23 HIE policy-related papers were reviewed out of which, 17 of them are national electronic health (eHealth) strategic documents [18–20,22,23,25–29,39–44], four were reports [12,21,24,45], and two were peer-reviewed papers [11,46]. The findings related to the national eHealth strategic documents were described in terms of architecture or framework development, policy or strategic objectives and policy or strategic initiatives while the findings related to reports and peer-reviewed papers were stated in terms of architecture adoption, policy and legislation recommendations related to interoperability and standards (Table 2).

**Table 2:** Architecture, strategic objectives, and strategic initiatives of reviewed papers.

Author,Year	Title, Country	Architecture	Strategic Objective	Strategic Initiative
FMOH, 2016	Information Revolution Roadmap, Ethiopia	Developed a National Health Information Enterprise Architecture that includes eHMIS and eLMIS, population data sources such as the census and surveys, and standards-based registries (Master Facility Registry and Data Dictionary that facilitate interoperability)	To establish an interoperable architecture to strengthen integration, standardization and harmonization among priority data sources and health information systems	-Develop and integrate national health information systems (eHMIS, eIFMIS, HGIS, eLIS, eRIS, eHRIS, HDD) -Develop and integrate standards-based digital registries (master facility registry and data terminology management service) -Develop and integrate point of service health information systems (Electronic Health Record (EHR), Terminology Management (TM), mHealth, and LMIS)
FMOH, 2017	eHealth Architecture, Ethiopia	Developed an architecture that enables: - client-level data exchange for health care facilities - aggregate data exchange to DHIS2 - Standardized data sharing	To develop interoperability functionality	**-**Identify and prioritize scenarios for data sharing -Instantiate a design for the interoperability layer - Identify risk matrix and subsequent risk mitigation plan - Pilot interoperability
			To identify and implement shared services consistent with eHealth architecture	**-** Identifying and supporting core information, data, and applications infrastructure - Identification and documentation of shared services - Development of shared services (master facility registry, national health data dictionary, and master person index)
			To identify and approve core interoperability standards	Identify core terminology and messaging standards
FMOH, 2018	Information revolution strategic plan, Ethiopia	Have eHealth Architecture	To improve standardization and integration of different HIS	-Develop and implement eHealth enterprise architecture -Develop and implement interoperability services -Implement a data warehouse hosted by the Ministry of Health (MOH) -Produce harmonized data dictionaries for the HMIS, disease classifications, and other priority datasets -Establish authoritative master health facility registry -Develop and implement shared health records -Initiate development of HIE (interoperability) standards.
MOH Rwanda, 2018	National Digital Health Policy, Rwanda	Developed an enterprise architecture that supports HIE	To strengthen integration and interoperability of HIS	Four initiatives were selected as flagship projects. The four initiatives envision standards for interoperability 1) Develop a health enterprise architecture that supports HIE. This initiative envisions • Updating the Rwanda Health Enterprise Architecture framework • Standards for interoperability • Policies for data privacy and exchange • Infrastructure at the National Data Warehouse to scale up the Health Information Exchange • Links to multi-sectoral initiatives including the “Citizen Service Bus” and/or direct links with Ubudehe and NID. Note: Rwanda was a pioneer among African countries in developing and enterprise architecture for health and implementing the first proof of concept for a health information exchange through the Rwanda Health Enterprise Architecture Project (2010–2014) 2) Implement EMR (nearly 400 health centers have openMRS-based HIV case management modules while 10 hospitals have a full package OpenMRS) 3) Developed citizen’s health portal that provide citizens with: • Access to their own personal history of health records • Information about health risks and real time data about disease trends • Tips for self-care–especially related to growing burden of on-communicable diseases (NCDs) • Information to help them find and select health care providers/services 4) Develop Telehealth
MOH Uganda, 2016	Uganda National eHealth Policy	eHealth enterprise architecture is taken as one of the national eHealth pillar	To establish eHealth enterprise architecture and interoperability framework and standards	- Plan, design and development eHealth Enterprise Architecture and Interoperability Framework to guide the implementation of all eHealth Services and Infrastructure in the country - Develop and implement compliance and review mechanism for the eHealth Enterprise Architecture and Interoperability Framework. - eHealth Services shall be established and implemented in line with the eHealth Enterprise Architecture and Interoperability Framework
MOH Kenya, 2016	Kenya National eHealth Policy 2016–2030	Set a policy priority to develop a national enterprise health systems architecture as a blueprint for design and implementation of eHealth systems	To enhance electronic exchange of health data and information	- Ensure standardization of stored data to promote interoperability of eHealth systems - Continuous improvement of infrastructure and resources to support cost-effective implementation of telehealth applications. - Ensure prompt and convenient access to patient’s demographic and clinicaldata to privileged healthcare providers.
			To develop standards and guidelines	- Develop EHR standards and guidelines - Develop Telemedicine standards and guidelines - Develop Interoperability Framework for eHealth systems and services; - Operationalize and implement eHealth Interoperability Standards and Guidelines; - Develop standards and guidelines for mHealth Systems to guide in the implementation
			To ensure interoperability and data interchange	- Operationalize and implement the Kenya Health Enterprise Architecture - Ensure functional interoperability - Ensure semantic interoperability
MOH Liberia, 2016	Liberian Health Information System and Information and Communication Technology (ICT) Strategic Plan 2016–2021	Set a strategic objective to develop HIS architecture, adopt an interoperability framework for the HIS system, and validated the architecture and disseminated SOP by end of 2017	To develop HIS architecture and adopt an interoperability framework for the HIS system	- Establish necessary software linkages and standardization of data sources for interoperability - Procure and establish necessary hardware and software for interoperability - Develop and validate a HIS architecture and interoperability roadmap and use case prioritization for system integration - Develop master facility registries and universal patient IDs to improve patient tracking - Identify areas for the use of appropriate mHealth interventions and develop a mHealth strategy and SOP for all levels
MOH Malawi, 2018	Monitoring, Evaluation, and Health Information Systems Strategy (MEHIS) 2017–2022	Set a strategic objective to develop an HIS enterprise architecture, interoperability requirements, harmonization of data elements and systems, and data exchange	To strengthen the interoperability of health information subsystems around a single country led platform	- Establish linkages between subsystems - Operationalize master health facility list - Operationalize terminology registry - Operationalize shared health record (patient-level central data repository) - Conduct migration of data from subsystems (e.g., logistics MIS and DHAMIS) into DHIS 2 while waiting for interoperability interface to be available
MOH Nigeria, 2015	National Health ICT Strategic Framework 2015–2020	Recommended a National Health ICT architecture that facilitates interoperability	To develop an architecture, standards and interoperability	- Define and implement a National Health ICT Architecture that defines high-level nationally-supported health information services - Implement and harmonize digital registries, data collection instruments and reporting indicators - Establish guidelines, minimum functional requirements, and interoperability standards
MOH Sierra Leone, 2018	National Digital Health Strategy 2018–2023, Sierra Leone,	Proposed the Asia health information network (AeHiN) enterprise architecture for Sierra Leone	To adopt architecture and standards framework	• Capacity building and standard adoption processes • Design architecture and standards to support interconnection (linkage) of patient, provider and health facility registries • Validate use-case(s) for which standards development will be based and align with available funding • Map and review relevant international standard in context of selected use-case(s) • Build capacity on identified standards • Model and adapt standards regime for selected use-case(s) • Document process and apply to other selected use case(s) • Develop relevant standards implementation guidelines per use-case • Create awareness on availability of use-case specific standards and advocate for more use-cases and standards • Advocate for digital health infrastructure • Assessment of status of current digital health infrastructure at health facilities
MOH SA, 2019	National Digital Health Strategy for South Africa 2019–2024	Have an architecture and framework for digital health interoperability but set a strategic objective to expand the framework and extend it into a health enterprise architecture	To establish an integrated information architecture for interoperability and effective, safe sharing of health information across health systems and services	- establish an open standards and open architecture approach - establish an Master Patient Index (MPI) for all South Africans - Implement HNSf conformance testing as a minimum for all health information systems. - Publish artefacts that allow users to easily adopt and implement standards. - Run interoperability hackathons to allow systems’ developers to test their systems and demonstrate interoperability. - All patient information systems to implement a unique identifier - Conceptualize and implement an integrated digital health platform. - establish the South African digital health platform. - establish a governance structure to reinforce digital health standards and interoperability. - Align the adoption of global standards with the South African Bureau of Standards (SABS)
MOH Tanzania, 2017	Tanzania Digital Health Investment Road Map 2017–2023	Set a recommendation to invest on the development of an enterprise architecture	To put in place an enterprise architecture, including governance, guidelines, and standards for interoperability	- develop a National eHealth standards framework - establish a governance framework for enterprise architecture - Enhance enterprise architecture capacity in the MOHCDGEC and PORALG - Software refinements to key systems to make them compliant with enterprise architecture
MOH Tanzania, 2019	The National Digital Health Strategy 2019–2024, Tanzania	There is an ongoing effort in developing a health enterprise architecture	To enhance seamless and secure information exchange	-Finalize and institutionalize Tanzania Health Enterprise Architecture - Strengthen use of data, application, and technology standards - Implement terminology services for standardized health terminologies, codes, data elements, and value sets - Strengthen interoperability across different systems within health and other sectors - Implement client and health worker registries - Strengthen the Health Facility Registry (HFR) and health commodities registries - Implement shared client health records - Strengthen standards and guidelines for secure data storage, processing, information exchange, and dissemination
MOH Uganda, 2017	Uganda National eHealth Strategy 2017–2021	Set a strategic objective to develop an eHealth Enterprise Architecture	To Plan, design and develop an eHealth Enterprise Architecture and Interoperability Framework	- Establish an eHealth Architecture and Interoperability Governance Structure - Conduct an eHealth Readiness Assessment Survey - Develop an eHealth Architecture and Interoperability development plan - Develop the eHealth Architecture and Interoperability Framework - Develop and implement National Health Information Exchange
			To Develop and Implement Compliance Assessment Mechanism to the eHealth Enterprise Architecture and Interoperability Framework	- Develop an eHealth Enterprise Architecture and Interoperability compliance framework - Certify all eHealth investments against the eHealth Architecture and Interoperability Framework - Set monitoring indicators, monitor compliance, report and act on violations
			To develop and Implement a Review Mechanism for the eHealth Enterprise Architecture and Interoperability Framework.	- Develop an eHealth Enterprise Architecture and Interoperability Review Mechanism - Review the framework after evaluating performance and in line with emerging requirements; - Revise the framework basing on the assessment
MOH Zambia, 2017	eHealth Strategy 2017–2021, Zambia	Set an objective to integrate and create an enterprise architecture	To foster integration and interoperability of eHealth systems	- Develop online master facility list for ease of citizen access - Establish an eHealth Coordination Team that will ensure eHealth system integration, interoperability, efficiency and sustainability across all eHealth projects. - Develop an electronic master patient index accessible to all eHealth systems, with a Unique ID. - Develop an electronic master drug listing available to all eHealth Systems. - Conduct mapping of all existing systems and functions. - Adopt standards for integration and interoperability. - Research and development of an interoperability layer. - Enforcement of interoperability and integration standards.
MOH Swaziland, 2016	Kingdom of Swaziland eHealth Strategy 2016–2020	eHealth architecture not mentioned	To identify, develop and/or adapt model eHealth solutions and best practices which, are integrated across health facilities in various tiers of the Swaziland health system.	- Develop Individual Electronic Health Information - Enhance Health Care Communications and Collaboration - Deploy Health Care Service Delivery Tools
			To develop, adopt and adapt, implement and continuously review national and international norms and standards to improve interoperability and the quality, safety and performance of eHealth practice in the country	Interoperability will be promoted across geographical areas within the country, programmatic areas, health care settings, and technology platforms
MOPH Cameroon, 2020	The 2020–2024 National Digital Health Strategic Plan, Cameroon	Absence of an HIS oriented architecture	To ensure the availability and application of ICT standards in 80% of health facilities at all levels of the health pyramid	Draft a standards framework.
			To ensure the interoperability of IT systems in 80% of health facilities at all levels of the health pyramid	- Ensure the secure exchange of data - Set up a software accreditation /certification system - Develop systems interoperability frameworks

Out of all eHealth strategic papers, four of them reported that their Ministry of Health has already developed an eHealth enterprise architecture that supports interoperability and standards for HIE [26,28,42,45]. One paper reported that there is an ongoing effort to develop an eHealth architecture [42] while another one already has an architecture for digital health but has a strategic plan to upgrade it to an eHealth enterprise architecture [26]. However, most of the papers (9) set a strategic objective to develop an eHealth enterprise architecture in their strategic plan [18,20–22,27,28,39,42]. The rest of the strategic papers reported the absence of eHealth architecture in their health system.

All the 17 reviewed strategic documents have at least one strategic objective in relation to establishing or implementing interoperability and standards for HIE. Three strategic papers set three strategic objectives related to architecture, interoperability and standards [18,20,40] while two strategic papers set two strategic objectives about interoperability and standards [27,29]. In addition to the nine strategic papers which set a strategic objective of developing an eHealth enterprise architecture, 10 papers were about the development of interoperability and standards for HIE [18,20,22,26–29,40,42,45], six papers about improvement [7,25,26,28,42,45], four papers about implementation [22,23,27,45], and three papers about adoption [23,27,40].

Several strategic initiatives were proposed by all the 17 strategic papers from African countries. Most of the initiatives include the development and implementation of eHealth architecture, the development and integration of various health information systems (e.g., Electronic Health Management Information System (eHMIS), Electronic Medical Record (EMR), Electronic Human Resource Information System (eHRIS), Electronic Logistic Information System (eLMIS), and Electronic Laboratory Information System (eLIS)) for HIE, development of standards for interoperability, preparation of health terminologies and data dictionaries for disease coding and classification, and establishment of master facility registry. Table 2 provides detailed information about the strategic papers including eHealth architecture, strategic objectives, and strategic initiatives.

Findings from the four reports and 11 peer-reviewed articles focused more on architecture adoption, policy and legislation recommendations related to interoperability and standards. A report in Liberia, for example, proposed the Open Health Information Exchange (OpenHIE) architecture (a registry-based model with federated systems) to coordinate specialized software solutions for specific subdomains within the Health Information System (HIS) [46]. Another report from South Africa recommended legislation, policy and compliance enabling the environment to achieve standards and interoperability [12]. A report from Digital Regional East Africa Community Health Initiative recommended shared standards for digital health that enable cross-border healthcare across the East Africa Community (ECA) region [43]. The recommendation includes developing and promoting regional principles for data sharing, system interoperability, and digital tool design. Another report from Digital Regional East Africa Community Health Initiative [44] forwarded the following strategic recommendation for ECA: 1) get political buy-in for new standards from partner States; 2) develop standards and protocols based on stakeholder inputs; 3) learn from other regions that have implemented shared standards and protocols; and 4) work closely with the legislation, policy, and compliance that impacts the development and compliance of interoperability standards.

In a peer-reviewed article from Botswana, legal, regulatory, and policy resolution of interoperability issues were raised as issues to be addressed [37]. In other peer-reviewed articles, four domains were suggested for interoperability framework implementation [11]. These are policy domain, governance and legal domain, organizational domain, document format, data modeling and coding domain, and data sharing domain.

### 3.5. HIE Standards

Of the 15 papers reviewed on HIE standards; four focused on designing a framework; two on developing a semantic interoperability platform, two on discussing health data exchange approaches and three focused on literature reviews on standard and interoperability of health information systems. In Kenya, Application Programming Interface (API)–Extensible Markup Language (XML) [31] was proposed to enable data interoperability. It was also recommended that a common concept dictionary based on the Columbia International eHealth Laboratory and the Millennium Villages Project (CIEL/MVP dictionary) should be developed to implement semantic interoperability [38]. A study explored the feasibility of HL7–XML to design a centralized medical informatics system for Ghana [30]. Another study recommended an architectural model for digital healthcare delivery that helps overcome integration and interoperability failures [36]. A study from Botswana [37] recommended the European eHealth Interoperability Framework (EIF) and the Australian eHealth Interoperability Framework. Similarly, a study in Africa proposed SIEMA architecture and Web services to improve data and semantic interoperability for a malaria surveillance system [33]. In Tanzania, data exchange components (DEC) recommended the realization of interoperability of electronic health records [32]. In 2018, Angula and Dlodlo proposed IHE and HL7, Enterprise Master Index, JavaScript Object Notation (JSON), Internet Control Message (ICMP) and Internet protocols for semantic interoperability of Namibia [34]. A study discussed how Health Level Seven Fast Healthcare Interoperability Resources (HL7 FHIR) based interoperability supported standards-based data exchange and interoperability between Electronic Medical Records and DHIS2 for Ethiopia, Kenya, Malawi and South Africa [47].

On the other hand, interoperability standards and health information architectures are included as a key priority and/or initiative in eHealth strategies of African nations (Ethiopia, Liberia, South Africa, Tanzania, Uganda and Zambia). The South Africa National Health Normative Standards Framework for interoperability sets a foundational base for interoperability [12]. In this strategic document, content standards (HL7 Clinical Document Architecture (CDA) and Continuity of Care Document (CCD)), identifier standards (International Standard Organization (ISO) 22220:2011 and ISO/TS 27527:2010), messaging standards (HL7 V2.X, Digital Imaging and Communications in Medicine (DICOM), Statistical Data and Metadata eXchange Data Management (SDMX-DM)), terminology standards (International Classification of Diseases (ICD)-10), procedure codes, medicine codes (Logical Observation Identifiers Names and Codes (LOINC) and Uniform Patient Fee Schedule (UPFS)) and content and structure standards (American Society Of Testing Materials (ASTM)/HL7 CCD, HL7 3 CDA, HL7 CRS), electronic health record standards (ISO/TR 20514:2005), health-specific security standards (ISO/TS 22600–1:2006, ISO/TS 22600–2:2006 and ISO/TS 22600–3:2009), a set of general standards and health-card standards were recommended for South Africa.

Tanzania, a leading African country to implement advanced health information exchange [17], set strategic initiatives to leverage digital health technologies. The initiatives were finalizing the Tanzania Health Enterprise Architecture, strengthening technology standards (ICD-10, HL7, DICOM, LOINC, and services codes), shared client health records and health facility registers. In addition, designing a national eHealth standards framework and governance framework for enterprise architecture were two of the 17 investment recommendations prioritized by the government of Tanzania to improve health system performance through better data use.

To foster health system integration in Zambia, the following strategies were set: developing a master facility file, electronic master patient index and International Standard protocols (HL7, ICD-10, International Classification of Primary Care (ICPC2), SNOMED and LOINC) and local standards from Zambia Information and Communications Technology Authority (ZICTA) [23]. In Ethiopia’s eHealth architecture, syntactic and semantic (such as ICD-10, LOINC, RxNorm, and SNOMED) standards were highlighted as basic standards [42]. Additionally, shared services such as Health worker registry, Master Facility Registry and National Health Data Dictionary (NHDD) to map the National Classification of Disease (NCoD) with ICD 10 were described in the architecture to support interoperability [42]. A study proposed HL7 FHIR, Aggregate Data Exchange (ADX), CSD, and ICD-10 as relevant standards for Liberia [48].

## 4. Discussion

A total of 32 papers were reviewed to explore the existing HIE policy and standards in African countries. Seventeen of them were HIE policy-related [4,11,18,19,21,22,24–27,29,39–41,43–45], nine were HIE standard-related papers [30–36,38,47], and six of them are both HIE policy and standard-related papers [20,23,28,37,42,46]. Results of the HIE policy review indicated that most of the papers recommended a strategic objective to develop an eHealth enterprise architecture that supports interoperability and standards [18,20–22,27,28,39,42]. Only a few governmental reports reported the presence of eHealth-related architecture [26,28,42,45]. E-health architecture helps as a framework not only for health information systems integration for information exchange but also a mechanism to avoid duplicate efforts in ICT health projects [49]. However, most African countries have not yet developed their eHealth architecture [18,20,22,25,40,43,46]. This could be due to the lack of ICT infrastructure and resources to develop integrated health information systems [50]. It seems that, however, African countries are recognizing the need to develop an eHealth architecture that enables interoperability and standards as witnessed by the strategic objectives of most African countries’ eHealth strategic plan.

The strategic objectives of the strategic documents discussed improvement, development, adoption, and implementation of eHealth architecture, interoperability and HIE standards [18,20–22,25–28,39,40,42,43,45,46]. This could indicate that most African countries will have an architecture that supports interoperability and standards for electronic HIE in the near future. Development and integration of health information systems were the main strategic initiatives forwarded by the Ministry of Health of African countries to achieve their strategic objectives [19,20,24,26–29,40,45,46]. African countries need to invest to have the necessary eHealth infrastructure that helps to smoothly integrate and run national health information systems.

Though attempts had been made, attaining standardized interoperable digital health solutions continues to be a daunting challenge, especially for Africa [51]. The way African countries approach the implementation of standards should not be from system silos to interoperable system silos. We found out that pilot HIE implementations across Africa were not guided by common data sharing standards and strategic policy directions except in Tanzania [28] and South Africa [7,12]. Though HIE often requires complex technical choices, the South Africa Normative Standards is a very good example to follow as a reference point [12]. Additionally, the move by Tanzania to deploy interoperable health systems following appropriate technical standardization has also been observed in our review [28]. In general, variations in relation to HIE policy and standards were observed in the different AU Member States. This implies the need for a continental policy and standard document that can serve as a guide for all African countries to exchange health information. The AU could take the lead in developing this HIE policy and standard document for the Member States.

## 5. Conclusion and Recommendation

African eHealth strategic documents and policies have paid attention to the development, improvement, adoption, and implementation of HIE architecture, interoperability, and standards for HIE. In addition, several strategic initiatives were in place. The common strategic initiatives are the development and integration of health information systems. This is a huge opportunity for Africans to have HIE across the continent. We recommend African countries have a common HIE policy that guides the integration of HIS across the continent to exchange health information through a common platform. Currently, there is an ongoing effort by the Africa CDC towards promoting HIE on the continent. A task force has been established from Africa CDC, HISP partners, and African and global HIE subject matter experts to provide expertise and guidance in the development of AU policy and standards for HIE. Although the work is still ongoing, the African Union shall continue to support the implementation of HIE policy and standards in the continent.

Synthetic and semantic interoperability standards were identified for the implementation of HIE in Africa. Our recommendations are for African countries and AU, and other regional bodies such as the Economic Community of West African States (ECOWAS), Common Market for Eastern and Southern Africa (COMESA), Southern African Development Community (SADC), Economic Community of Central African States (ECCAS), EAC (East Africa Community), Intergovernmental Authority on Development (IGAD), etc. For African countries, we recommended that comprehensive interoperable technical standards should be set at a national level and should be guided by appropriate governance and legal framework, data ownership and use agreements and health data privacy and security guidelines. Establishing a governance structure to reinforce digital health system interoperability was mentioned as one strategic initiative in Tanzania, South Africa and Uganda’s digital health strategies. Similarly, to facilitate HIE in and out of African countries, there is a need to define the interoperability governance framework that serves as a guiding principle for HIE in Africa.

On the other hand, HIE legal framework is important to ensure all the HIE policies and standards, and applicable state and local laws and regulations, including, but not limited to, protecting the confidentiality and security of health information. Additionally, African countries need to enter into data use and data sharing agreements. Establishing the privacy and security protocols, understanding the current security conditions and solutions, and selecting the appropriate patient identification and authentication standards are initiatives that need to be considered across Africa. On top of the policy issues, African nations need to identify a set of standards (health system standards, communication, messaging standards, terminology/vocabulary standards, patient profile standards, privacy and security, and risk assessment) and implement them throughout all levels of the health system. Many of the existing standards will likely be updated and or superseded over time; however, a set of standards that support interoperability should be stable and revised only when it is needed [48]. On top of this, we recommend that the AU and regional bodies provide the necessary human resource and high-level technical support to African countries to implement HIE policy and standards.

## 6. Limitation

This study has a set of limitations. First, we used only 5 databases and 1 eHealth strategic document repository. Using other databases and/or repositories may result in additional studies and a broader reflection of HIE in Africa. Second, we only considered articles published in English. Other important publications written in other languages might be missed. Third, gray literature such as websites, blogs, poster sessions and others were not reviewed in our study and only peer-reviewed publications, secondary analyses, thesis, and eHealth strategic documents of selected African countries were used. Fourth, the heterogeneity between peer-reviewed articles and national eHealth strategic documents made the synthesis of the reported literature daunting and difficult which could limit the possibility of identifying hidden themes.

## Supporting information

S1 File Appendix A: Critical Appraisal. Appendix B: Data Extraction from Peer-Reviewed Papers. Appendix C: Data Extraction from Strategic Documents and Government Reports. Appendix D: Acronyms and Abbreviation
(DOCX)
