# Supplementary material for: Health information exchange policy and standards for digital health systems in africa: A systematic review
Source: PLOS Digit Health. 2022 Oct 10;1(10):e0000118. doi: 10.1371/journal.pdig.0000118 (PMC9931258; doi:10.1371/journal.pdig.0000118)
Supplement: S1 File — Appendix A: Critical Appraisal. Appendix B: Data Extraction from Peer-Reviewed Papers. Appendix C: Data Extraction from Strategic Documents and Government Reports. Appendix D: Acronyms and Abbreviation (DOCX) [file pdig.0000118.s001.docx]

**[Appendix](https://www.ihe.net/) A: Critical Appraisal**

| **Author** | **Year** | **Problem Statement** | **Objective** | **Method** | **Citation** | **Discussion** | **Result usefulness** | **Result applicability** | **Quality** |
| --- | --- | --- | --- | --- | --- | --- | --- | --- | --- |
| Damoah D., et al | 2014 | Well written | Not clear | Literature review | No citations are used but references are listed | No | Useful | Applicable | Low |
| Keny A, et al | 2015 | Well stated | Clear | Stated clearly | Yes | Yes | Useful | Applicable | High |
| Oluoch T, et al | 2015 | Well stated | Clear | Observational study | Yes | Yes | Useful: HIE implementation shown | Applicable: HIE implementation shown | High |
| Adenuga OA, et al | 2015 | Well stated | Clear | Proposed architecture based on Literature review | Yes | Yes | Result not clearly demarcated but useful | Not applicable because only eHealth architecture shown although the architecture support interoperability | Low |
| Ndlovu K, et al | 2016 | Oversimplified | Clear | Literature review | Yes | Yes | Useful. Results are based on description of Botswana’s eHealth systems | Applicable: interoperability of eHealth systems in Botswana are shown | Low |
| Brenas JH, et al | 2017 | Well stated | Clear | Design and development of the semantic interoperability | Yes | Description of Architecture which supports interoperability | Useful. A framework that support interoperability is discussed | Applicable: Architecture/Framework is described | Moderate |
| Chali F, et al | 2018 | Well stated | Clear | Survey and Architecture design for data exchange | Yes | Yes | Useful | Applicable | High |
| Angula N and Dlodlo N | 2018 | Well stated | clear | Design and development of framework for disease surveillance data semantic interoperability | Yes | No discussion was made. Just description of the findings | Useful | Applicable | Moderate |
| Angula N and Dlodlo N | 2018 | Well stated | Clear | Case study and design and development of semantic interoperability framework | Yes | No discussion was made. Just description of the findings | Useful | Applicable | Moderate |
| Stroetmann KA | 2019 | Well stated but not structured | Clear | Survey, case studies, and literature review | Yes | Yes | Useful | Applicable | Moderate |
| Baskaya M, et al | 2019 | Well stated | Clear | Implementation of HL7 for interoperability | Yes | Result and discussion together | Useful | Applicable | High |

**Appendix B- Data Extraction from Peer-Reviewed Papers**

| **Author, Year** | **Objective** | **Design** | **HIE policy** | **HIE Standard** |
| --- | --- | --- | --- | --- |
| Tom Oluoch, 2015 | To assess the effect of interoperable national eHIS on enhancing data availability in Kenya | Developed system interoperability environment |  | API – XML |
| Karl A. Stroetmann, 2019 | To sketch the core steps necessary to develop and implement digital health ecosystem, and to specify fundamental building blocks. | Literature Review | - Interoperability framework is identified as very important in the implementation of Digital Health Ecosystem which is identified as one of the four foundational building blocks for implementing a comprehensive Digital Health Ecosystem.  - Five domains are suggested for the interoperability framework: Policy domain, Governance and legal domain, Organizational domain, and Document format, data modelling and coding domain | - |
| Keny A, et al, 2015 | To describe an approach, taken by Kenya, to come up with a national level concept dictionary to serve multiple HER implementations across the country | Discussion by ‘Concept Working Group’ | - | -Kenya Common Concept Dictionary  recommended based on the dictionary maintained by Columbia  International eHealth Laboratory and the Millennium Villages  Project (CIEL/MVP dictionary)  - can be mapped to SNOMED CT, LOINC, ICD-10, RxNORM and CVX |
| Damoah et al., 2014 | To explored the feasibi1ity of implementing and deploying a centralized medical informatic s system for Ghana using health 1eve1 7 (HL7) message standard. | System design and development | - | -HL7 messages in XML (Extended Markup Language) format implemented |
| Adenuga et al., 2015 | To propose eHealth  architectural model for healthcare delivery for developing countries | Literature Review | - | Architectural model |
| Ndlovu et al., 2016 | -To outline global and local endeavors towards the attainment of interoperable eHealth solutions  -To suggest a possible eHealth interoperability framework direction for developing economies such as in Botswana | Literature Review | - legal and regulatory issues, policy and resolution  of interoperability raised as issues to be addressed | -European eHealth Interoperability Framework (EIF) and Australian eHealth Interoperability Framework were recommender |
| Brenas et al., 2017 | - to introduce a framework in which access to distributed data repositories containing malaria-related  - to facilitate uninterrupted dynamic surveillance queries across multiple resources | Framework development | - | - SIEMA architecture with presentation tier, a query tier, a service tier and a data access tier developed.  -Web services for Semantic interoperability  - HTTP used to establish communication challenge among components  - SPARQL querying purpose  - Semantic Automatic Discovery and  Integration (SADI) designing I/O  - Rule Interchange Format (RIF) for rule mapping |
| Chali et al., 2017 | -To offers a mechanism to integrate a single mobile application with multiple electronic health records (eHRs) while mitigating the interoperability problem  - To support the on-going efforts to alleviate the interoperability problem of eHRs by designing and implementing a data exchange mechanism that leverages mobile devices deployed in healthcare. | System design and development techniques used | **-** | **-** data exchange component (DEC) to realize interoperability of eHRs without specific data exchange standards |
| Angula and Dlodlo, 2018a | - To design and develop a framework for data semantic interoperability of DHIS and these other health information silo systems | qualitative approach (case study) and Design and develop a framework | **-** | -IHE and Health Level Seven (HL7) are implemented to interface DHIS and silo systems  - Enterprise Master Index |
| Angula and Dlodlo, 2019 | -to design and develop a framework for data semantic interoperability of DHIS and these other health information silo systems | qualitative approach (case study) and Design and develop a framework | **-** | -JSON  -internet control message (ICMP-IP) protocols |
| Baskaya et al., 2019 | -To provides insights into how mHealth4Afrika is leveraging HL7 FHIR to support standards-based data exchange and interoperability between Electronic Medical Records and DHIS2. | Discussion about a specific platform- not clear | **-** | -HL7 FHIR based  interoperability |

**Appendix C- Data Extraction from Strategic Documents and Government Reports**

| **Author** | **Year** | **Problem Statement** | **Objective** | **Method** | **Citation** | **Discussion** | **Result usefulness** | **Result applicability** | **Quality** |
| --- | --- | --- | --- | --- | --- | --- | --- | --- | --- |
| FMOH, Ethiopia | 2017 | Well stated | clear | No | No proper citation | Description of the proposed system | Useful. | Applicable | Moderate |
| FMOH, Ethiopia | 2018 | Well stated | clear | No | No proper citation | Description of the proposed system | Useful. | Applicable, policy for stds and integration of HIS described | low |
| FMOH, Ethiopia | 2016 | Well stated | clear | No | No proper citation | Description of the proposed system | Useful. | Applicable, policy exist | low |
| Leitner C, et al | 2016 | Well stated | clear | Yes, described | Yes | No | Useful. | Applicable, HIS interoperability status described. Interoperability options for different HIS proposed | high |
| CSIR and NDoH, SA | 2014 | Well stated | clear | Yes, In-depth interview, use cases developed | Yes | Description of the results | Useful. | Applicable, sets of interoperability standards are identified, implementation guideline developed | high |
| MOH Rwanda | 2018 | Well stated | clear | Yes, situation analysis performed | Yes | Description of the results | Useful. | Applicable, HIE in Rwanda is developed and implemented, piloted in Rwamagana. Policy set to strengthen integration and interoperability of HI | high |
| MOH, Uganda | 2016 | Well stated | clear | Yes, situation analysis performed | Yes | Description of the results | Useful. | Applicable, eHealth Enterprise Architecture and Interoperability Framework and Standards are touched but not described in detail | Moderate |
| MOH, Kenya | 2016 | Well stated | clear | Yes, situation analysis performed | Yes | Description of results | Useful | Applicable: set policy to recognize the need for eHIE. Policy is set that forces all systems to be integrated with existing DHIS2, integrated human resource information system, LMIS… | Moderate |
| MOH, Liberia | 2016 | Well stated | clear | Yes, assessment strategy and development process described | No | Description of results | Useful | Applicable: set strategies to integrate various HIS and propose interoperable HIS architecture in Annex K | Low |
| MOH, Malawi | 2018 | Well stated | clear | Yes, situational analysis | No | Description of results | Useful | Applicable: set policy and planned to strengthen interoperability of HIS around a single country led platform, and set as a deliverable to have an interoperable system. | Moderate |
| MOH, Nigeria | 2015 | Well stated | clear | Yes, situational analysis | No | Description of results | Useful | Applicable: developed a national HI architecture (interoperability architecture) and have policy regarding architecture, standards, and interoperability | Moderate |
| MOH, Sierra Leone | 2018 | Well stated | clear | Yes, situational analysis | Yes | Description of results | Useful | Applicable: architecture and standard framework for interoperability developed and budget set | Moderate |
| MOH, SA | 2019 | Well stated | clear | Yes, situational analysis | Yes | Description of results | Useful | Applicable: set a policy or a plan to establish an integrated architecture for interoperability, put strategic intervention for standards and interoperability | Moderate |
| MOH, Tanzania | 2017 | Well stated | clear | Yes, situational analysis | Yes | Description of results | Useful | Applicable: set plan to put in place an enterprise  Architecture for standards and interoperability | Moderate |
| MOH, Tanzania | 2019 | Well stated | clear | Yes, situational analysis | Yes | Description of results | Useful | Applicable: set a strategic initiative of strengthening interoperability across different HIS and prepared a roadmap | Moderate |
| MOH, Uganda | 2017 | Well stated | clear | Yes, situational analysis, methodology to develop eHealth strategy is stated. | Yes | Description of results | Useful | Applicable: set a pillar/policy to develop an eHealth enterprise architecture, interoperability and standards | Moderate |
| MOH, Zambia | 2017 | Well stated | clear | Yes, situational/ SWOT analysis performed | No | Description of results | Useful | Applicable: set a policy/plan to develop a national HIE in consultation with … set an objective to foster integration and interoperability of eHealth systems | Low |
| East Africa Research Group Commission | 2017 | Well stated | clear | No | No | Description of results | Useful | Applicable: set a policy and region and partner state responsibilities for harmonization, standards and interoperability | Low |
| East Africa Research Group Commission | 2018 | Well stated | clear | No | No | Description of results | Useful | Applicable: set an implementation approach to identify and develop standards, and protocols to lay the foundation for regional digital health ecosystem by developing standards and interoperability. | Low |
| MOH, Cameron | 2020 | Well stated | clear | Yes, situational analysis performed | No | Description of results | Useful | Applicable: Standards and interoperability are set as a strategic area. major components of an ecosystem of interoperability and standards are identified | Medium |
| MOH, Swaziland | 2016 | Well stated | clear | No | Yes | Description of results | Useful | Applicable | Low |

**Appendix D: Acronyms and Abbreviation**

ADX	Aggregate Data Exchange

API	Application Programming Interface

Africa CDC	[Africa Centres for Disease Control and Prevention](https://www.devex.com/organizations/centres-for-disease-control-and-prevention-africa-cdc-120577)

ASTM	American Society of Testing Materials

AU	Africa Union

CCD	Continuity of Care Document

CDA	Clinical Document Architecture

COMESA	Common Market for Eastern and Southern Africa

COVID-19	Coronavirus Disease 2019

CRS	Care Record Summary

CSD	Care Service Discovery

DHIS	District Health Information Software

DICOM	Digital Imaging and Communications in Medicine

EAC	East Africa Community

ECOWAS	Economic Community of West African States

ECCAS	Economic Community of Central African States

eHealth	Electronic Health

eHMIS	Electronic Health Management Information System

EHR	Electronic Health Record

eHRIS	Electronic Human Resource Information System

eLMIS	Electronic Logistic Information System

eLIS	Electronic Laboratory Information System

EMBASE	Excerpta Medica Database

EMR	Electronic Medical Record

FHIR	Fast Healthcare Interoperability Resources

FMOH	Federal Ministry of Health

HL7	Health Level Seven

HL7 FHIR	Health Level Seven Fast Healthcare Interoperability Resources

HIS	Health Information System

HISP	Health Information Service Provider

ICD	International Classification of Diseases

ICPC2	International Classification of Primary Care

IGAD	Intergovernmental Authority on Development

ICT	Information and Communication Technology

IHE	Integrating the Healthcare Enterprise

ISO	International Organization for Standardization

JSON	JavaScript Object Notation

LOINC	Logical Observation Identifiers Names and Codes

MEDLINE	Medical Literature Analysis and Retrieval System Online

mHealth	Mobile Health

MOH	Ministry of Health

MPI	Master Patient Index

NCDs	Non-communicable diseases

NCoD	National Classification of Disease

NHDD	National Health Data Dictionary

OpenHIE	Open Health Information Exchange

OpenMRS	Open Medical Record System

SA	South Africa

SADC	Southern African Development Community

SDMX	Statistical Data and Metadata eXchange

SDMX-DM	Statistical Data and Metadata eXchange Data Management

SIEMA	Semantic Interoperability & Evolution for Malaria Analytics

SNOMED	Systematized Nomenclature of Medicine

TM	Terminology Management

UPFS	Uniform Patient Fee Schedule

WHO	World Health Organization

XML	Extensible Markup Language

[ZICTA](https://www.ihe.net/)	Zambia Information and Communications Technology Authority
